# The Multi-Fungicide Resistance Status of *Aspergillus fumigatus* Populations in Arable Soils and the Wider European Environment

**DOI:** 10.3389/fmicb.2020.599233

**Published:** 2020-12-15

**Authors:** Bart Fraaije, Sarah Atkins, Steve Hanley, Andy Macdonald, John Lucas

**Affiliations:** ^1^NIAB, Cambridge, United Kingdom; ^2^Rothamsted Research, Harpenden, United Kingdom

**Keywords:** *Aspergillus fumigatus*, antifungal resistance, azoles, MBC fungicides, QoI fungicides, SDHI fungicides, environment

## Abstract

The evolution and spread of pan-azole resistance alleles in clinical and environmental isolates of *Aspergillus fumigatus* is a global human health concern. The identification of hotspots for azole resistance development in the wider environment can inform optimal measures to counteract further spread by minimizing exposure to azole fungicides and reducing inoculum build-up and pathogen dispersal. We investigated the fungicide sensitivity status of soil populations sampled from arable crops and the wider environment and compared these with urban airborne populations. Low levels of azole resistance were observed for isolates carrying the CYP51A variant F46Y/M172V/E427K, all belonging to a cluster of related cell surface protein (CSP) types which included t07, t08, t13, t15, t19, and t02B, a new allele. High levels of resistance were found in soil isolates carrying CYP51A variants TR_34_/L98H and TR_46_/Y121F/T289A, all belonging to CSP types t01, t02, t04B, or t11. TR_46_/Y121F/M172V/T289A/G448S (CSP t01) and TR_46_/Y121F/T289A/S363P/I364V/G448S (CSP t01), a new haplotype associated with high levels of resistance, were isolated from Dutch urban air samples, indicating azole resistance evolution is ongoing. Based on low numbers of pan-azole resistant isolates and lack of new genotypes in soils of fungicide-treated commercial and experimental wheat crops, we consider arable crop production as a coldspot for azole resistance development, in contrast to previously reported flower bulb waste heaps. This study also shows that, in addition to azole resistance, several lineages of *A. fumigatus* carrying TR-based CYP51A variants have also developed acquired resistance to methyl benzimidazole carbamate, quinone outside inhibitor and succinate dehydrogenase (Sdh) inhibitor fungicides through target-site alterations in the corresponding fungicide target proteins; beta-tubulin (F200Y), cytochrome *b* (G143A), and Sdh subunit B (H270Y and H270R), respectively. Molecular typing showed that several multi-fungicide resistant strains found in agricultural soils in this study were clonal as identical isolates have been found earlier in the environment and/or in patients. Further research on the spread of different fungicide-resistant alleles from the wider environment to patients and *vice versa* can inform optimal practices to tackle the further spread of antifungal resistance in *A. fumigatus* populations and to safeguard the efficacy of azoles for future treatment of invasive aspergillosis.

## Introduction

*Aspergillus fumigatus* is a mold commonly found on plant debris and in soil. It is also an opportunistic human pathogen causing allergic symptoms and life-threatening invasive infections. The incidence of invasive aspergillosis (IA) has been increasing in recent years largely due to increased numbers of immunocompromised individuals in the population unable to fight off infection. Treatment of IA is difficult as there are few effective antifungal drugs without toxic side-effects. Azoles are among the most widely used antifungals due to their efficacy and low toxicity. However, resistance to azoles has occurred in clinical isolates of *A. fumigatus* and is becoming more common, with potentially serious consequences for the treatment of invasive infections.

The first cases of azole resistance in *A. fumigatus* were reported in clinical strains from the USA isolated during the late 1980's (Denning et al., 1997). Recent studies have shown that resistance to medical azoles in both clinical and environmental isolates has increased in Europe and elsewhere since the late 1990's (Snelders et al., 2008; Howard et al., 2009). Azoles inhibit the enzyme sterol 14α-demethylase (CYP51), a key step in the synthesis of sterols essential for the integrity of cell membranes. *Aspergillus fumigatus* has two CYP51 proteins, CYP51A and CYP51B. Many different resistant strains, mostly with CYP51A alterations, have been isolated from patients undergoing azole therapy (Howard et al., 2009). Other resistance mechanisms have also been found, including increased expression of *CYP51* (Camps et al., 2012a,b; Buied et al., 2013) and efflux pump encoding genes (Fraczek et al., 2013; Meneau et al., 2016), accumulation of ergosterol precursors (Hagiwara et al., 2018; Rybak et al., 2019), and reduced intracellular retention of azoles (Wei et al., 2017).

Highly azole-resistant isolates have been found in the Netherlands in azole-naïve patients since 2007 (Van der Linden et al., 2011). These clinical strains and the majority of azole-resistant environmental isolates that have been characterized belong to two unique genotypes based on a combination of CYP51A alterations and promoter tandem repeat (TR) inserts of 34 or 46 bp. Clinical isolates carrying simultaneously CYP51A alterations TR_34_ and amino acid substitution L98H (TR_34_/L98H) have been found in Europe since 1998 (Snelders et al., 2008; Lazzarini et al., 2016), whereas the first TR_46_/Y121F/T289A isolate was reported from North America in 2008 (Wiederhold et al., 2016). These genotypes, which are now spread worldwide Verweij et al., 2016), are also highly resistant to several azole fungicides commonly used to preserve materials (e.g., wood, paints, and fabrics) and to prevent fungal diseases in animals, birds and plants. Subsequently, concerns have been raised about an environmental route of resistance selection through an unintended exposure of *A. fumigatus* as a non-target pathogen to azole fungicides in agricultural settings (Verweij et al., 2009; Berger et al., 2017; Hollomon, 2017). Detailed information on the origin and further spread of pan-azole resistant strains in the wider environment is therefore urgently needed (Chowdhary and Meis, 2018). This will enable thorough assessment of the extent of risk as predicted by Gisi (2014), and implementation of strategies to slow down and/or prevent future spread of azole resistance.

Flower bulb waste, green waste and wood chippings have recently been reported as “hotspots” for azole resistance development in the Netherlands (Schoustra et al., 2019). A hotspot is defined as a habitat that supports the growth and reproduction of *A. fumigatus* for relatively long periods of time in the presence of azole residues at concentrations that can select for resistant strains. This aim of this study is to investigate if fungicide applications on arable crops, particularly cereals, can be regarded as a hotspot for azole resistance development. Large amounts of fungicides are used to control diseases in cereals, including several triazoles that have been shown to have similar CYP51 binding modes and high levels of cross-resistance to medical azoles (Snelders et al., 2012). As such, living in agricultural areas was suggested to increase the risk of inhaling azole-resistant isolates (Rocchi et al., 2014). However, due to a low competitive ability of *A. fumigatus* to grow on straw in comparison with other saprophytic fungi, the low residue levels and short periods of bioavailability of azoles in soils after foliar spray applications, the risk of azole resistance development is estimated to be low (Gisi, 2014). Previous studies carried out with a limited number of samples and isolates suggest pan-azole resistance can be detected at low frequencies (2–3%) in UK cereal fields (Bromley et al., 2014; Tsitsopoulou et al., 2018).

Having access to the long-term Park Grass (permanent grassland since 1856, no fungicides), as well as Broadbalk (continuous winter wheat since 1843) and two experiments examining the effects of repeatedly incorporating straw of continuous wheat crops at Rothamsted and Woburn with plots that have been sprayed with foliar fungicides or left untreated (Macdonald et al., 2018, enabled us to investigate if cereal foliar fungicide applications can select for resistance in *A. fumigatus* populations. For comparison, we also isolated and characterized *A. fumigatus* strains from air samples and soils of arable crops sampled at different locations in Europe using cell surface protein (CSP) sequence analysis (Klaassen et al., 2009) and microsatellite typing based on short tandem repeats (STR*Af* ) (De Valk et al., 2005). The majority of strains were not only tested for sensitivity to several clinical and agricultural azoles but also to fungicides with different modes of action, including methyl benzimidazole carbamate (MBC), quinone outside inhibitor (QoI) and succinate dehydrogenase inhibitor (SDHI) fungicides, targeting beta-tubulin, cytochrome *b* and succinate dehydrogenase (Sdh) subunits B, C, and D, respectively. These fungicides are all commonly used to control diseases in arable crops and horticulture. An improved understanding of resistance development to different classes of fungicides in the environment can provide more information on the emergence and origin of novel resistant genotypes, and where and under which circumstances, selection is likely to occur in environmental and/or clinical settings.

## Materials and Methods

### Sampling of Soils

Soil from Park Grass and some sections of Broadbalk have never been exposed to azole fungicides. Soils from other sections of Broadbalk have been annually exposed to single or multiple azole treatments since 1979. The amounts and identity of azoles used in seed treatments and foliar sprays reflect commercial practices and are recorded each year in the Results of the Classical and other Long-term Field Experiments available via the electronic Rothamsted Archive (http://www.era.rothamsted.ac.uk/eradoc/book). During 2012–2015, prothioconazole was used in seed treatments and three azoles were applied in foliar sprays that were applied as part of a three-spray based disease management programme using mixtures of fungicides belonging to different mode of actions. Epoxiconazole, nine out of 12 applications, was most often used, followed by tebuconazole and prothioconazole, which were used three times together in a spray application. For this study, we sampled soil from plot 3d (permanent pasture, receiving no fertilizers or chalk inputs) on Park Grass and from strips 2.2 (farmyard manure), 3 (no fertilizers), and 8 (144 kg N, 90 kg K, and 12 kg Mg per hectare) on sections 1 (with spring and summer fungicide treatments) and 6 (no fungicide treatments) under continuous winter wheat on Broadbalk (Macdonald et al., 2018). Soils were sampled to a depth of 5 cm using a 3 cm diameter auger at three sampling points, separated five meters apart, in July 2015. Samples of air-dried topsoil (0–23 cm) collected in August 2016 were also available from three replicated plots without straw incorporation (straw removed) and from three replicated plots in which fungicide-exposed straw was incorporated at four times the annual straw yield on the Long-term Amounts of Straw Experiments at Rothamsted and Woburn (Macdonald et al., 2018). These experiments examined the effects of long-term straw incorporation on soil properties and yields of continuous wheat grown on contrasting soils at Rothamsted and Woburn (silty clay loam v sandy loam). They began in 1987 and were stopped after 30 years (Powlson et al., 2011). The azole fungicides used in these experiments, as part of mixtures with other fungicides belonging to different modes of action, during 2013–2016 are listed in Table 1. The wheat seeds grown on these experiments were also treated with prothioconazole before drilling. Topsoil to a depth of 5 cm was also sampled from 15 commercial wheat fields in Germany (locations Burscheid, Vechta, Göttingen, Dormagen, and Ergolding), France (Tierce, Obenheim, Grisolles, Lignon, and Reims), and the UK (Kent, Suffolk, Somerset, Norfolk, and Herefordshire). In addition, topsoils representing arable crops, woodland, and grass verges were also sampled from 14 other locations in five countries across Europe (Table 2). Each topsoil sample contained three subsamples, each collected at three different sampling points separated at least 5 m apart.

**Table 1:** The long-term amounts of straw experiments at Rothamsted and Woburn.

**Location/foliar azole exposure**	**Straw incorporation/plot numbers**	**Frequency pan-azole resistant strains per plot^a^**
**Rothamsted farm**	Nil (straw removed)	
Epoxiconazole (2013, 2014, 2015, 2016)	1	0/7
Prochloraz (2014, 2016)	8	1/18
Prothioconazole (2013, 2015, 2016)	12	0/10
Tebuconazole (2013, 2014, 2015, 2016)	Four times amount of straw	
	3	1/16
	5	0/14
	9	0/19
**Woburn farm**	Nil (straw removed)	
Epoxiconazole (2013, 2014, 2015, 2016)	19	1/6
Prochloraz (2015)	21	0/8
Prothioconazole (2014, 2015, 2016)	28	1/10
Tebuconazole (2014, 2015, 2016)	Four times amount of straw	
	20	0/5
	23	0/4
	27	0/5

*Foliar exposure to azoles and the frequency of pan-azole resistant A. fumigatus isolates in soils sampled in 2016*.

a *Pan-azole resistant (R) strains have elevated MIC levels for voriconazole (>1.0 μg/ml), imazalil (>2.5 μg/ml), and tebuconazole (>3.0 μg/ml)*.

**Table 2:** Soil samples from different geographical regions sampled in 2015 and the frequency of pan-azole resistant *A. fumigatus* strains.

**Sample**	**Location**	**Soil description**	**Number of pan-azole resistant strains^a^**
SS1	Penzesgyör, Hungary	Wheat field	0/10
SS2	Vönock, Hungary	Sunflower field	0/10
SS3	Vönock, Hungary	Woodland	0/10
SS4	Kenyeri, Hungary	Corn field	0/14
SS5	Kemmelbach, Austria	Grass verge at petrol station	0/12
SS6	Hunderdorf, Germany	Grass verge at petrol station	0/10
SS7	Hösbach, Germany	Ploughed cereal field	0/16
SS8	Waremme, Belgium	Sugar beet field	1/10
SS9	Adinkerke, Belgium	Sugar beet field	0/10
SS10	Afferden, The Netherlands	Corn field	1/12
SS11	Beekbergen, The Netherlands	Forest	0/10
SS12	Boxtel, The Netherlands	Forest	0/10
SS13	Boxtel, The Netherlands	Harrowed sugar beet field	0/10
SS14	Boxtel, The Netherlands	Corn field	0/10

a *Pan-azole resistant (R) strains have elevated MIC levels for voriconazole (>1.0 μg/ml), imazalil (>2.5 μg/ml) and tebuconazole (>3.0 μg/ml)*.

### Sampling of Airborne *A. fumigatus* Strains

Airborne spores were captured on untreated or fungicide amended Sabouraud dextrose (SD) agar (Oxoid Ltd, Basingstoke, UK) containing penicillin (100 U/ml) and streptomycin (100 μg/ml) using mobile spin air samplers (IUL, Spain). Each time, 5,000 L of air was sampled during rotating of SD agar plates at 1 rpm for 50 min. Fungicide amended agar plates contained carbendazim (10 μg/ml), pyraclostrobin (10 μg/ml) or tebuconazole (5 μg/ml). Colonies of *A. fumigatus* were recovered from the plates after two days incubation at 48°C.

### Isolation of *A. fumigatus* Strains and Inoculum Preparation

To isolate strains belonging to the *A. fumigatus* complex, 2 g aliquots of soil samples were added to 8 ml of phosphate buffered saline amended with 0.1 % (v/v) Tween 20. After 2 h incubation at 37°C with shaking (150 rpm), the supernatant after sedimentation was plated out on SD agar amended with penicillin (100 U/ml) and streptomycin (100 μg/ml). Colonies of *A. fumigatus* were recovered from the plates after 2 days incubation at 48°C. No or low numbers up to 10 colony forming units per g soil were usually detected. After subculturing single colonies in tissue culture flasks with 12 ml SD agar for seven days at 37°C, spores were harvested through shaking with 5 mm glass beads after addition of 3 ml of saline. Final spore suspensions were directly used for storage in 50 % (v/v) glycerol at −80°C, DNA extractions or for fungicide sensitivity testing using spiral plating.

### Fungicide Sensitivity Testing Using Spiral Plating

Spore suspensions containing ~10^6^ spores/ml in sterile distilled water were used in the microprocessor controlled Autoplate Spiral Plating System AP5000 (Advanced Biosystems) according to the manufacturer's instructions. This method has been used for antimicrobial susceptibility testing of fastidious bacteria and fungi (Förster et al., 2004; Pong et al., 2010). The test fungicides, solutions made in DMSO, were placed in a sample cup of the spiral plater and automatically plated at exponentially decreasing concentrations achieving. Depending on the molecular weight of the compounds, an up to a 200-fold fungicide dilution gradient on SD agar was achieved using 15 cm plates. The concentration ranges (μg/ml) for the different fungicides were: boscalid (0.1–18.469), carbendazim (0.1–11.464), imazalil (0.25–43.153), itraconazole (0.025–6.113 or 0.1–22.324), pyraclostrobin (0.1–20.120), tebuconazole (0.1–17.349), terbinafine (0.01–1.7), and voriconazole (0.1–19.120). The different concentration ranges were chosen to distinguish sensitive wild-type (wt) isolates without known resistance mechanisms with those of insensitive isolates harboring resistance mechanism in one assay. Isolates were streaked on these spiral SD agar plates (8 per plate) from the outside to the center using cotton swaps and incubated at 37°C in the dark. After 24 h incubation, the fungal growth of each isolate on the spiral plate was visually assessed and the MIC value determined using the Spiral Gradient Endpoint (SGE) software.

### DNA Extractions

After harvesting spores from one-week cultures in tissue culture flasks, 1.5 ml of spore suspensions was transferred into a 2 ml tube and centrifuged for 2 min at 13,200 rpm. After removing the supernatant, DNA was extracted according to the MasterPure Yeast DNA Purification kit (Lucigen Corporation) with the inclusion of an extra bead-beating step which involved the addition of glass beads (0.425–0.600 mm) and the use of the Genie 2 Vortex (Scientific Industries) at full power for 2 min. This step was carried out after the lysis step just before adding the MPC Protein Precipitation Reagent.

### PCR Amplification and Sequencing

All PCR reactions were carried out using the Easy A cloning Enzyme kit (Agilent Technologies, UK). Typical reactions of 40 μl contained 4.0 μl Easy A cloning buffer (10 x stock), 0.8 μl dNTPs (10 mM stock), 30.4 μl PCR grade water, 0.2 μl of each of primer (100 μM primer stocks) (Supplementary Table 1), 0.4 μl Easy A cloning enzyme, and 4.0 μl genomic DNA (40 ng total). PCR cycling started with an initial denaturation of 95°C for 2 min, followed by 40 cycles of denaturation (10 s at 95°C), annealing (20 s at annealing temperature) and extension (depending on amplicon size 1 or 2 min at 72°C), and a final extension (8 or 9 min at 72°C) and hold step at 4°C. All primers and corresponding annealing temperatures are shown in Supplementary Table 1. PCR products were visualized by agarose gel electrophoresis to confirm the expected PCR amplicon size, and subsequently sent to MWG Eurofins (UK) for purification and sequencing using the primers used in PCR or with additional primers when needed (Supplementary Table 1). Primers used in this study were either reported before or designed based on published *A. fumigatus* sequences for CYP51A (Genbank Accession JX283445), CYP51B (AF338660), beta tubulin (NC_007200; region 70221-71948), cytochrome *b* (JQ346808), and succinate dehydrogenase subunit B (NC_007194.1; region 2654821-2655836), C (NC_007198.1; region 2501949-2502752), and D (NC_007198.1; region 4215045-4215968) covering all regions of the fungicide target encoding genes where mutations affecting inhibitor binding have been reported (Mair et al., 2016). Sequences were analyzed and aligned using Geneious software version 10.0 (Biomatters, New Zealand).

### Short Tandem Repeat and Cell Surface Protein Typing

Isolates of *A. fumigatus* with different levels of azole sensitivity were further characterized using microsatellite genotyping based on a panel of nine short tandem repeat markers (STR*Af* 2A, 2B, 2C, 3A, 3B, 3C, 4A, 4B, and 4C) according to the method previously described and validated (De Valk et al., 2005; De Groot and Meis, 2019). In addition, the cell surface protein (CSP) (XM_749624.1) encoding gene of *A. fumigatus* was also partially sequenced from selected strains (Balajee et al., 2007). CSP typing of strains was carried out according to the nomenclature proposed by Klaassen et al. (2009), which is based on the tandem repeat region, in which up to 10 different 12-bp repeat sequences have been found in different copy numbers, and the flanking regions. After manual alignment of sequences, a phylogenetic tree for the different CSP sequences encountered in this study was constructed using the Geneious Tree Builder software (Biomatters, New Zealand) based on the Tamura-Nei distance model and the Neighbor-Joining method.

## Results

### Isolation and Fungicide Sensitivity Testing of *A. fumigatus* Isolates From the Broadbalk and Park Grass Long-Term Experiments

**Figure 1:**
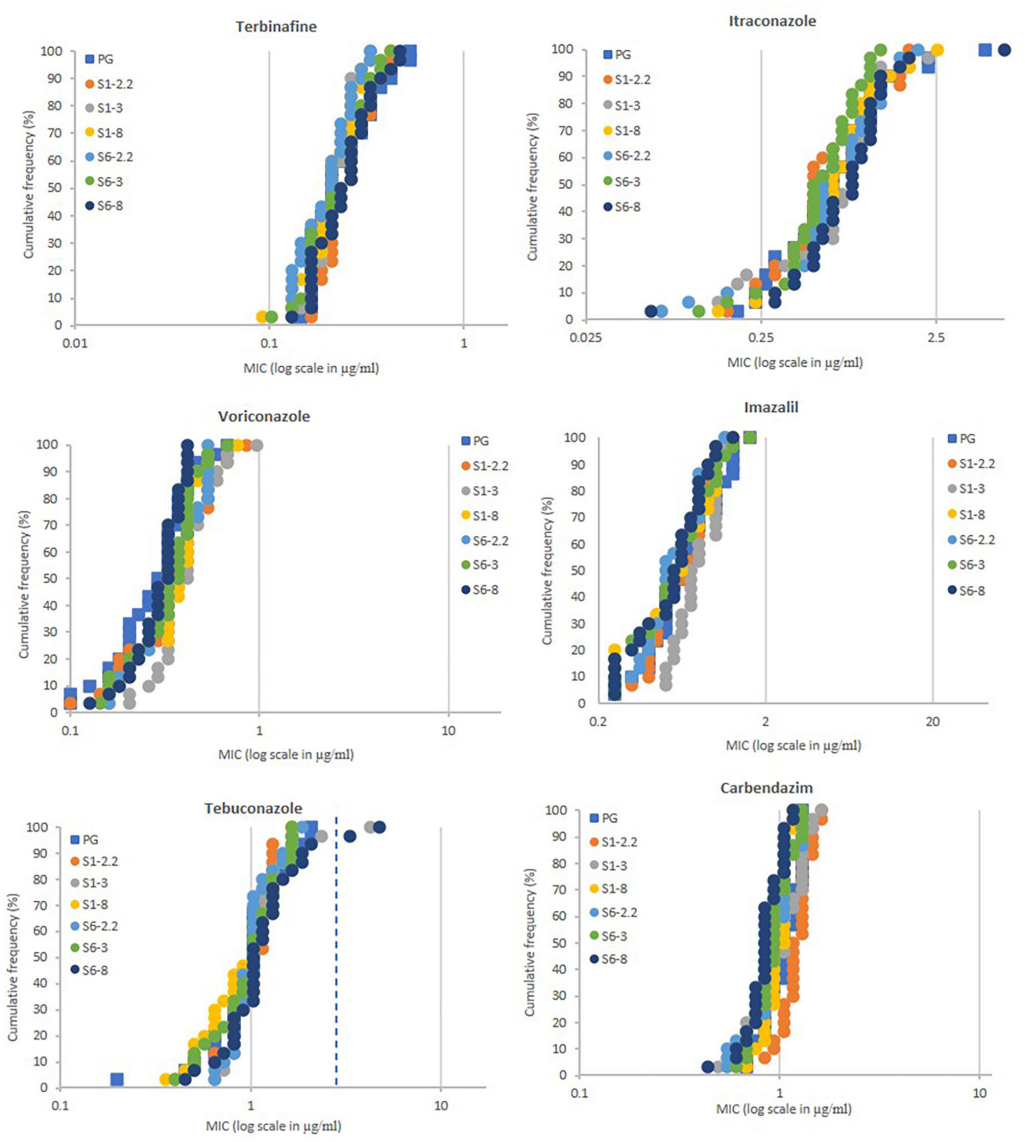
Azole sensitivity profiling of *A. fumigatus* populations (each *n* = 30) sampled from soils of the Broadbalk (continuous wheat) and Park Grass (permanent grass land) long-term experiments at Rothamsted. MIC values of individual isolates (X-axis in μg/ml) are plotted against the proportion of the population (Y-axis in %). PG, Park Grass, S1 (section 1 with fungicides at Broadbalk), S6 (section 6 without fungicides at Broadbalk) with treatments 2.2 (farmyard manure), 3 (untreated) and 8 (standard fertilizers). MIC values (ppm) for highly resistant isolates to itraconazole (>6.113) and sensitive to voriconazole (<0.1) and imazalil (<0.25) are displayed as 6.113, 0.1, and 0.25, respectively. Extra dotted line for tebuconazole shows cut-off value of 3.0 ppm.

A total of 180 *A. fumigatus* strains were isolated from soils taken from selected plots on the Broadbalk Wheat Experiment, at Rothamsted (Harpenden, Hertfordshire, UK) (Macdonald et al., 2018). Soils were sampled from plots receiving three different fertilizer/manure treatments [plots 2.2 (farm-yard manure), 3 (nil fertilizers), and 8 (mineral fertilizers)] within each of two sections under continuous winter wheat, grown with (section 1) and without (section 6) spring or summer fungicides, but including fungicide seed treatments. In addition, 30 strains were isolated from soils taken from the Park Grass Continuous Hay Experiment (permanent grass land, plot 3d - no liming and fertilizers) at Rothamsted (Macdonald et al., 2018). MIC testing using spiral plating showed for the wild-type (WT-NL), TR_34_/L98H (TR34-NL), and TR_46_/Y121F/T289A (TR46-NL) reference strains the following average values in μg/ml: 0.338, 0.180, and 0.286 for terbinafine, 1.056, >6.113, and >6.113 for itraconazole, 0.342, 1.505, and >19.120 for voriconazole, 0.978, 3.156, and >43.153 for imazalil, 0.829, 6.876, and >17.349 for tebuconazole, and 1.029, 1.048, and >11.464 for carbendazim, respectively. High levels of resistance to all four azoles and, unexpectedly, carbendazim were measured for the reference strain TR46-NL. Except for itraconazole, strain TR34-NL showed more moderate levels of resistance to azoles in comparison with strains WT-NL and TR46-NL, but similar levels of sensitivity to terbinafine were recorded in all reference strains. The Broadbalk and Park Grass isolates showed no significant differences in the sensitivity levels between the six different populations tested, with most isolates showing similar levels of control as the wild-type reference strain in typical dose-response curves (Figure 1). The dynamic range was 0.092 – 0.53, 0.059 – 4.757, 0.1 – 0.965, <0.249 – 1.622, 0.198 – 3.341, and 0.433 – 1.453 μg/ml for terbinafine, itraconazole, voriconazole, imazalil, tebuconazole and carbendazim, respectively. Only two strains, sampled from section 1 plot 3 (isolate BB1-3-B9) and section 6 plot 8 (isolate BB6-8-C7) showed resistance to itraconazole (MICs >6.113 μg/ml) and tebuconazole (MICs of 4.227 and 4.755 μg/ml) but not to voriconazole (MICs of 0.675 and 0.418 μg/ml) and imazalil (MICs of 1.015 and 0.714 μg/ml) in the first screen.

### Isolation and Fungicide Sensitivity Testing of *A. fumigatus* Isolates From the Long-Term Amounts of Straw Experiments at Rothamsted and Woburn

**Figure 2:**
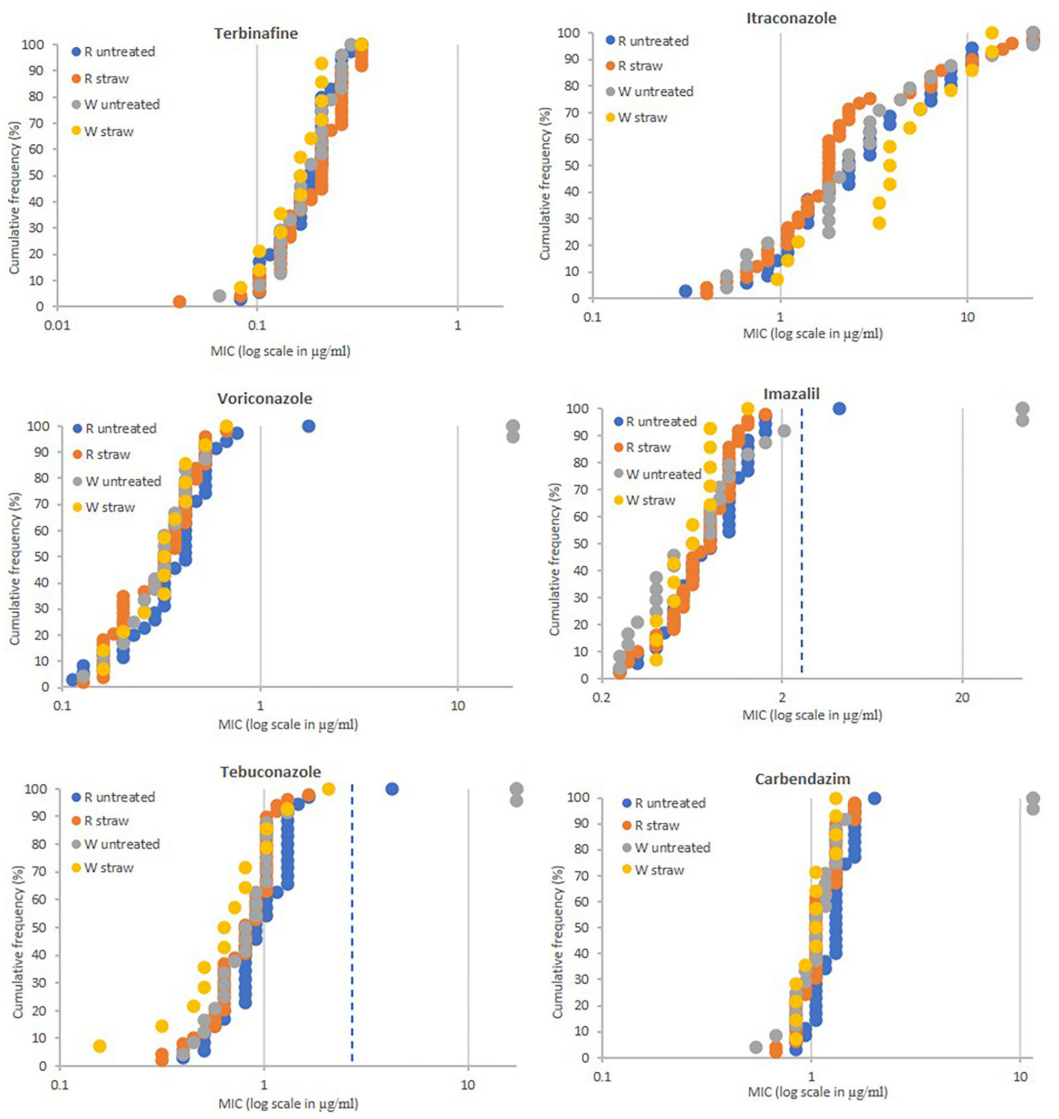
Fungicide sensitivity profiling of *A. fumigatus* populations sampled from soils of the Long-term Amounts of Straw Experiments at the Rothamsted (*n* = 84) and Woburn farms (*n* = 38). MIC values of individual isolates (X-axis in ppm) are plotted against the proportion of the population (Y-axis in %). Populations from Rothamsted (R) with straw incorporation (straw) (*n* = 49) and without straw (untreated) (*n* = 35) and Woburn (W) with straw incorporation (straw) (*n* = 14) and without straw (untreated) (*n* = 24). MIC values (μg/ml) for highly resistant isolates to itraconazole (>22.324), voriconazole (>19.120), imazalil (>43.153), tebuconazole (>17.349), and carbendazim (>11.464), and sensitive to imazalil (<0.25) are displayed as 22.324, 19.120, 43.153, 17.349, 11.464, and 0.25, respectively. Extra dotted line for imazalil and tebuconazole show cut-off values of 2.5 and 3.0 ppm, respectively.

In total 84 strains were isolated from the wheat straw incorporation experiment at Rothamsted, 35 came from three control plots (plots 1, 8, and 12) and 49 from three plots where straw was incorporated (plots 3, 5, and 9) (Table 1). The soils sampled at the Woburn Experimental Farm (Woburn, Bedfordshire) contained less strains and only 24 and 14 strains were isolated from untreated (plots 19, 21, and 28) and straw incorporated plots (20, 23, and 27), respectively (Table 1). Fungicide sensitivity was carried out as before except itraconazole, for which a higher spiral plate concentration range of 0.1–22.324 μg/ml was used in subsequent studies. The sensitivity profiles were similar for all populations tested showing no differences between untreated and straw incorporated plots (Figure 2). A significant number of isolates, 18 out of 122 tested, were highly resistant to itraconazole with MIC values >10 μg/ml. Only four isolates, three from untreated plots (two at Woburn and one from Rothamsted), and one from a straw incorporated plot at Rothamsted, showed moderate to high levels of resistance to voriconazole, imazalil, and tebuconazole using cut-off MIC values of 1.0, 2.5, and 3.0 μg/ml, respectively. Three of these isolates, RS3-3, WN19-3, and WN28-6, showing the highest MIC for all four azoles tested (all out of range), were also highly resistant to carbendazim with MIC values >11.464 μg/ml, which is similar to the profile of the TR_46_/Y121F/T289A reference strain. A lower level of azole resistance was measured for the carbendazim sensitive isolate RN18-8 which has a similar sensitivity profile to the TR_34_/L98H reference strain. All strains tested were sensitive to terbinafine, with MIC values between 0.041 and 0.322 μg/ml.

### Isolation and Fungicide Sensitivity Testing of *A. fumigatus* Isolates From Soils of Commercial Wheat Fields in Germany, France, and the UK

**Figure 3:**
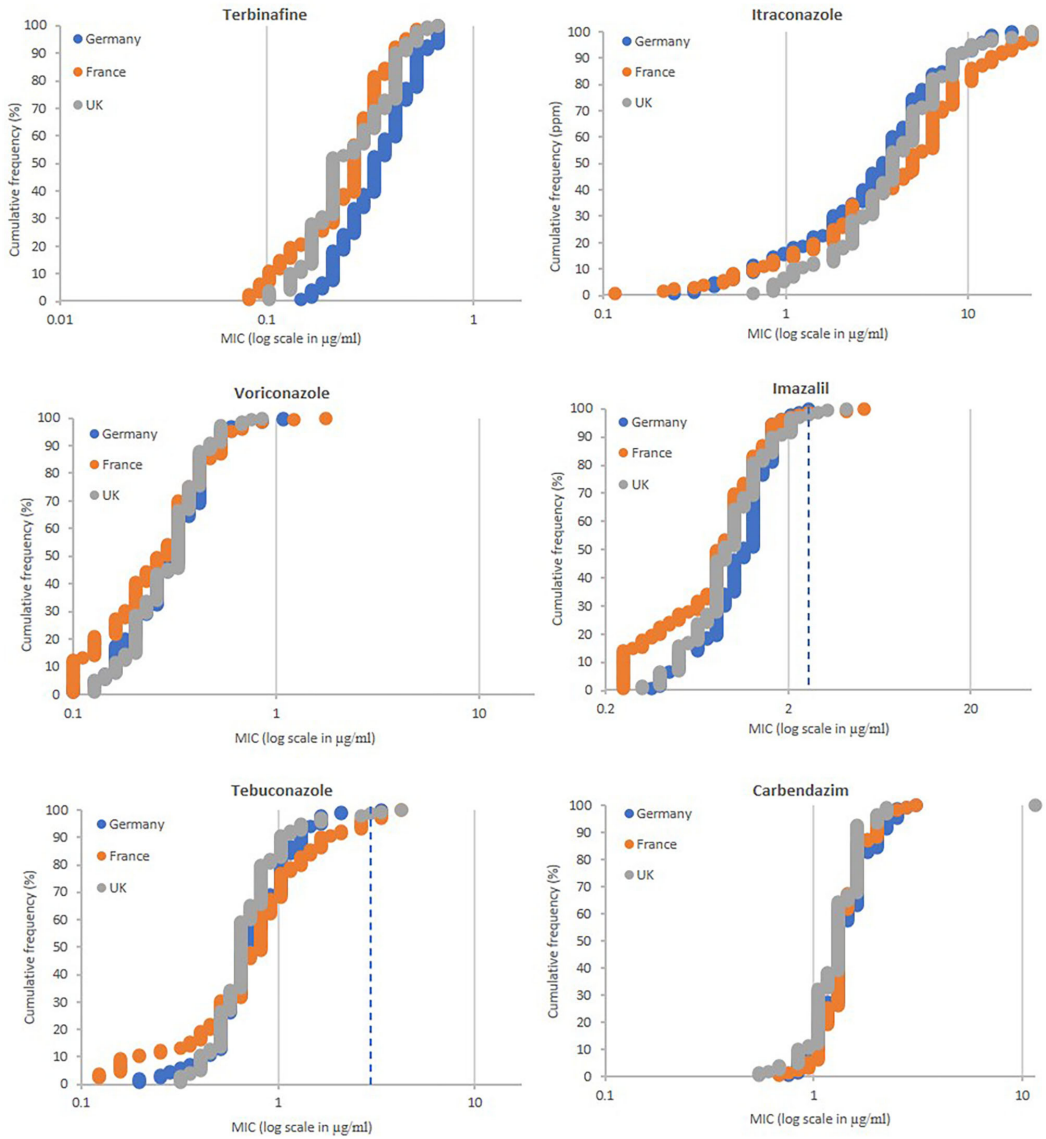
Fungicide sensitivity profiling of *A. fumigatus* populations sampled from soils of commerial wheat fields in Germany (*n* = 149), France (*n* = 129), and the UK (*n* = 140). MIC values of individual isolates (X-axis in ppm) are plotted against the proportion of the population (Y-axis in %). MIC values (ppm) for highly resistant isolates to itraconazole (>22.324) and carbendazim (>11.464) are displayed as 22.324 and 11.464, respectively. MIC values (μg/ml) for highly resistant isolates to itraconazole (>22.324), voriconazole (>19.120), imazalil (>43.153), tebuconazole (>17.349), and carbendazim (>11.464), and sensitive to voriconazole (<0.1) and imazalil (<0.25) are displayed as 22.324, 19.120, 43.153, 17.349, 11.464, 0.1, and 0.25, respectively. Extra dotted line for imazalil and tebuconazole show cut-off values of 2.5 and 3.0 ppm, respectively.

In total 428 strains were isolated, of which 149, 139 and 140 came from Germany (locations Burscheid, Vechta, Göttingen, Dormagen, and Ergolding) France (Tierce, Obenheim, Grisolles, Lignon, and Reims) and the UK (Kent, Suffolk, Somerset, Norfolk, and Herefordshire), respectively. Thirty strains were isolated from all locations with exception of Ergolding (29), Grisolles (29), Reims (10 because subsamples were pooled), and Suffolk (20, only two subsamples available). The fungicide sensitivity profiles of the populations were similar for all locations tested (Figure 3). All strains were sensitive to terbinafine having MIC values between 0.082 and 0.669 μg/ml. A high number of isolates, 12 from Germany, 25 from France and 11 from the UK, showed MIC values >10 μg/ml for itraconazole. Only a few strains showed low levels of resistance to voriconazole, imazalil and tebuconazole using cut-off MIC values of 1.0, 2.5, and 3.0 μg/ml, respectively. Regarding voriconazole, only two strains from Germany (G1-A1 and G1-A9 from Burscheid) and two from France (F1-C5 from Tierce and F5-B5 from Reims) showed low resistance levels with MICs between 1.0 and 2.0 μg/ml. Two, three and four strains from Germany, France and the UK, respectively, were moderately resistant to imazalil with MICs between 2.5 and 5.0 μg/ml. One, five and two strains from Germany, France and the UK, respectively, were moderately resistant for tebuconazole with MICs between 3.0 and 6.0 μg/ml. None of the strains tested were resistant to all four azoles tested, but isolates UK5-B5 (Herefordshire, UK) and F1-C5 (Tierce, France), both highly resistant to itraconazole (MIC >22.324 μg/ml) were the only strains showing resistance to two out of the three other azoles tested. UK5-B5 was the only strain with resistance to carbendazim (MIC >11.464 μg/ml).

### Isolation and Fungicide Sensitivity Testing of *A. fumigatus* Isolates From Soils at Different Locations in Europe

Strains of *A. fumigatus* were isolated from soils sampled at 14 different locations in the Netherlands, Belgium, Germany, Austria and Hungary (Table 2). Ten or more strains per sample (154 strains in total) were further tested for sensitivity to terbinafine, itraconazole, voriconazole, imazalil, tebuconazole, and carbendazim (data not shown). All isolates were sensitive to terbinafine and carbendazim. Ten strains from locations in Hungary (Kenyeri), Austria (Kemmelbach), Germany (Hösbach), Belgium (Waremme), and the Netherlands (Afferden) showed itraconazole MICs >10.0 μg/ml, but only two of those strains, SS8-7 from a sugar beet field near Waremme in Belgium, and SS10-6 from a corn field near Afferden in the Netherlands, showed voriconazole MICs >1.0 μg/ml and were also less sensitive to imazalil and tebuconazole with MICs just above 2.5 and 3.0 μg/ml, respectively. Two strains from a sugar beet field near Adinkerke (Belgium) showed only a slightly raised MIC value of 3.341 μg/ml for tebuconazole. All other strains were sensitive to all azoles tested.

### Isolation and Fungicide Sensitivity Testing of *A. fumigatus* Isolates From Aerosols

During 11–14 February 2018, 26–29 May 2018, and 17–20 May 2019, airborne spores were captured in Boskoop (the Netherlands), Boxtel (the Netherlands), or Harpenden (UK) on untreated and fungicide amended Sabouraud dextrose agar plates from air volumes of 5,000 L using mobile spin air samplers (IUL, Spain). Assuming an adult air intake of 14,000 L, the daily exposure to *A. fumigatus* varied between 14 and 193 conidia in Boskoop under cold and wet conditions in the afternoon on 11 February 2018 and in Boxtel during the evening on 20 February 2019, respectively. Only a few colonies were growing fast on tebuconazole amended plates on several occasions, which was compared to the recorded colony numbers on untreated plates equivalent to frequencies of up to 4%.

Five isolates, captured on carbendazim amended agar in Boxtel (BTCa-1) and Boskoop (BKCb-1), tebuconazole amended agar in Boxtel (BTTa-1) and pyraclostrobin amended agar in Harpenden (HPPb-1 and HPPb-2) in February 2018 were further tested for fungicide sensitivity. All five were highly resistant to both carbendazim and pyraclostrobin with MICs >11.464 and >20.120 μg/ml, respectively. BTTe-1 and BKCb1 were also highly resistant to imazalil (MIC >43.153 μg/ml), voriconazole (MIC >19.120 μg/ml), and tebuconazole (MIC >17.349 μg/ml), similar to the profile of the TR_46_/Y121F/T289A reference strain. BTCa-1, HPPb-1, and HPPb-2 showed low to moderate levels of resistance to two or more of the azoles tested. All strains tested were sensitive to terbinafine with MICs between 0.116 and 0.595 μg/ml.

### Cell Surface Protein Typing of *A. fumigatus* Strains Isolated From the Long-Term Broadbalk and Park Grass Experiments

**Figure 4:**
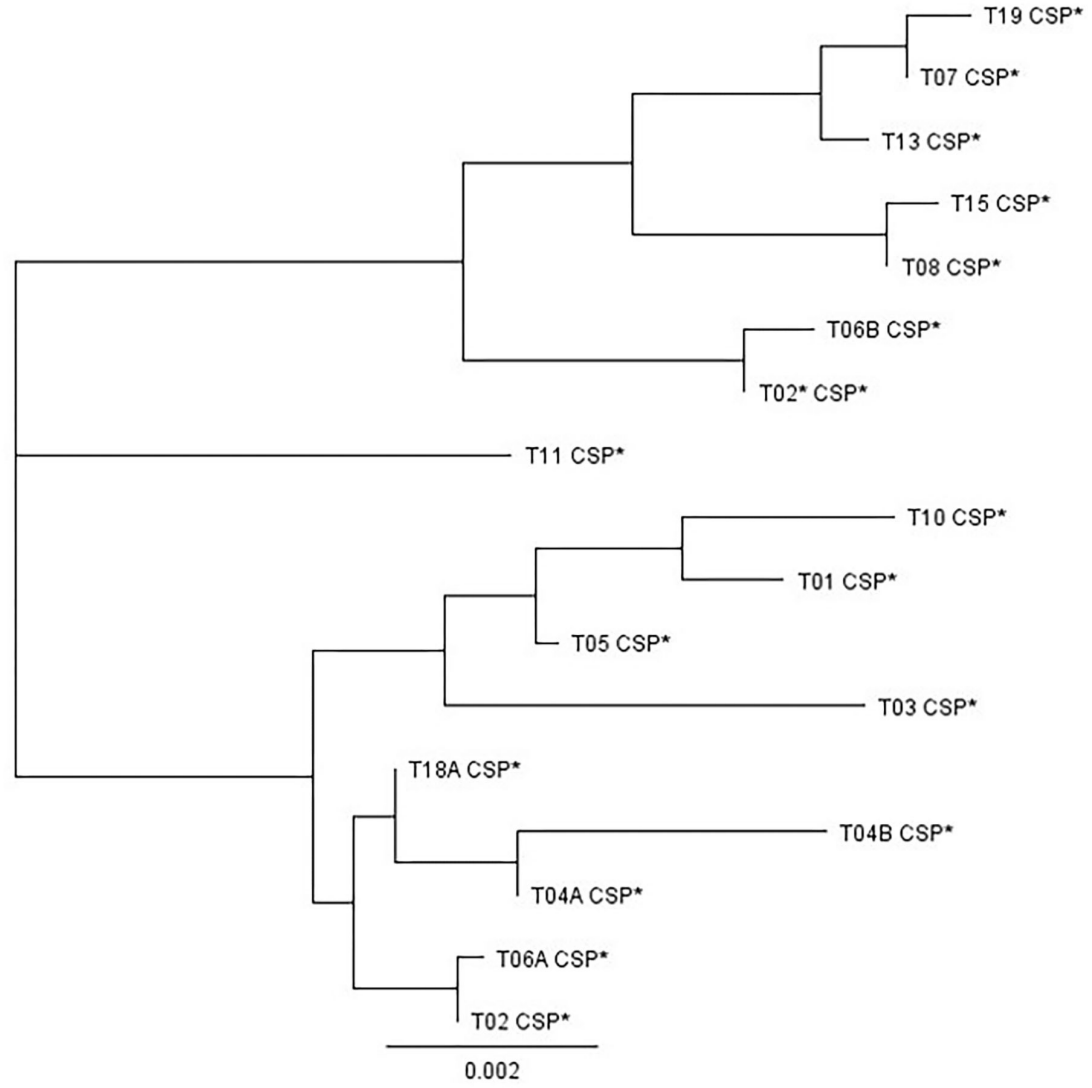
Phylogenetic clustering of the different CSP variants found in this study. The sequences used in this analysis covered the variable tandem repeat region and flanking regions of – 221 bp and + 6 or + 9 bp, depending on each CSP type. CSP t15 and t19, containing 16 successive 12-bp tandem repeats and an extra codon (+ 9 bp flanking region) produced the largest fragment of 422 bp. The shortest sequence was obtained for t03, 311 bp, with seven successive 12-bp tandem repeats and without extra codon (+ 6 bp flanking region).

A selection of isolates, 58 in total, was further characterized using cell surface protein (CSP) PCR amplicon sequencing (see primers in Supplementary Table 1). Figure 4 shows the relatedness amongst the different CSP types identified in this study. Of the 57 strains tested, 14 belonged to CSP type t03, 11–t04A, 9–t18A, but they all came exclusively from a farm yard manure treated plot, 8–t02, 7–t01, 4–t05, 2–t06A, 2–t11, and one each to t08, t19 and a new CSP type. The new CSP type was named as t02^*^ because the same tandem repeat succession as type t02 was found, but with changes in the flanking regions of the tandem repeat region at codon−14 (*C*TC instead of GTC), +1 (CC*G* instead of CCA), and +3 (CCT duplication) (Kidd et al., 2009). The itraconazole insensitive Broadbalk strains BB1-3-B9 and BB6-8-C7 belonged to CSP t02^*^ and t19, respectively. When additional itraconazole insensitive isolates from commercial wheat fields in Germany, France and the UK were tested, a high proportion belonged to CSP t08, followed by t13, t15, t19, t02^*^, and t07 (data not shown). All these CSP types cluster together and have also a SNP at codon−55 of the flanking region (TG*T* instead of TGC) in common (Figure 4).

### Azole Resistance Phenotype-to-Genotype Relationship of Isolates

A selection of 30 azole insensitive and sensitive isolates, including six older reference strains, were further characterized using an additional genotyping assays and phenotyping screens (Tables 3, 4). Cross-resistance was observed for all four azoles tested, especially between voriconazole, tebuconazole and imazalil (Table 4). For itraconazole, two strains (BTTa-1 and BKCb-1) highly insensitive for voriconazole, tebuconazole and imazalil showed only moderate levels of insensitivity to itraconazole, whereas several strains sensitive or moderately insensitive to voriconazole, tebuconazole, and imazalil (e.g., UK2-B4 and G1-A9) were highly insensitive to itraconazole. CYP51A sequencing showed a clear pheno-to-genotype trend, with TR_46_/Y121F/T289A, TR_46_/Y121F/M172V/T289A/G448S, and TR_46_/Y121F/T289A/S363P/I364V/G448S isolates showing high levels of insensitivity to imazalil, voriconazole and tebuconazole (Table 4). Mutations leading to S363P (serine (TCT) replaced by proline (*C*CT) at codon 363) and I364V (isoleucine (ATT) replaced by valine (*G*TT) at codon 364) have not been reported before (see GenBank MW119308). Lower levels of insensitivity were measured for TR_34_/L98H strains, while some strains carrying F46Y/M172V/E427K showed insensitivity to tebuconazole and/or itraconazole with MIC values >3.0 and 10 μg/ml, respectively. D262Y was the only mutation found in sensitive strains. CYP51B sequencing of isolates BB1-3-B9, RN8-18, RS3-3, and WN19-9 revealed no mutations.

**Table 3:** Origin and characterization of *Aspergillus fumigatus* isolates using CYP51A sequencing, CSP typing, and STR*Af* profiling.

**Isolate^a^**	**Origin/year**	**CYP51A**	**CSP**	**STR*****Af*** **marker profile**								
				**2A**	**2B**	**2C**	**3A**	**3B**	**3C**	**4A**	**4B**	**4C**
WT-NL	Netherlands, before 2014	Wt	t01	-	-	-	-	-	-	-	-	-
PG3-3	UK, 2015	Wt	t01	27	18	15	8	11	31	26	10	8
AF65	UK, 1997	Wt	t02	14	20	12	35	9	10	8	10	21
PG2-10	UK, 2015	Wt	t04B	18	12	8	27	10	18	9	9	5
BB1-2.2-B1	UK, 2015	Wt	t18A	13	21	11	31	27	8	10	9	8
BB6-8-B1	UK, 2015	D262Y	t03	20	19	8	36	14	22	9	9	5
SS5-2A	Austria, 2015	D262Y	t05	18	12	9	10	10	12	8	10	7
PG2-6	UK, 2015	D262Y	t05	15	20	9	10	10	6	8	10	10
BB1-3-B9	UK, 2015	F46Y/M172V/E427K	t02^*^	10	14	10	17	13	8	7	5	6
G1-A9	Germany, 2016	F46Y/M172V/E427K	t02^*^	10	14	10	17	13	8	7	5	6
SS5-7C	Austria, 2016	F46Y/M172V/E427K	t08	10	16	10	17	13	20	7	5	6
UK2-B4	UK, 2016	F46Y/M172V/E427K	t08	10	15	10	26	11	8	7	5	5
F5-C6	France, 2016	F46Y/M172V/E427K	t15	10	14	10	17	13	13.1	7	5	5
BB6-8-C7	UK, 2015	F46Y/M172V/E427K	t19	10	15	10	19	12	12	7	5	6
AF293	UK, 1993	F46Y/M172V/N284T/ D255E/E427K	t06A	26	18	18	46	21	23	11	10	8
TR34-NL	Netherlands, before 2014	TR_34_/L98H	-	-	-	-	-	-	-	-	-	-
UK5-B5	UK, 2016	TR_34_/L98H	t02	14	20	8	31	9	10	8	10	11
BTCa-1	Netherlands, 2018	TR_34_/L98H	t02	-	-	-	-	-	-	-	-	-
HPPb-1	UK, 2018	TR_34_/L98H	t02	25	20	18	31	9	10	10	14	5
08-19-02-10	Netherlands, 2008	TR_34_/L98H	t04B	25	24	12	84	9	9	8	10	11
RN8-18	UK, 2016	TR_34_/L98H	t04B	25	24	12	84	9	9	8	10	11
SS10-6A	Netherlands, 2016	TR_34_/L98H	t11	20	21	16	16	12	7	16 3	24	33
SS8-7A	Belgium, 2016	TR_34_/L98H	t11	20	21	16	77	12	11	16 3	11	21
F1-C5	France, 2016	TR_34_/L98H	t11	-	-	-	-	-	-	-	-	-
BTTa-1	Netherlands, 2018	TR_46_/Y121F/M172I/ T289A/G448S	t01	26	21	12	26	10	6	13	9	20
TR46-NL	Netherlands, before 2014	TR_46_/Y121F/T289A	-	-	-	-	-	-	-	-	-	-
WN19-3	UK, 2016	TR_46_/Y121F/T289A	t01	10	19	12	45	9	50	8	10	10
WN28-6	UK, 2016	TR_46_/Y121F/T289A	t01	20	20	14	49	9	36	8	9	9
RS3-3	UK, 2016	TR_46_/Y121F/T289A	t02	10	20	8	42	9	10	12	10	20
BKCb-1	Netherlands, 2018	TR_46_/Y121F/T289A/ S363P/I364V/G448S	t01	26	20	12	43	11	11	12	9	9

*Isolates grouped according to CYP51A sequence and CSP type*.

a *AF65 and AF293 are clinical isolates; -, unknown or not determined*.

**Table 4:** CYP51A variants and sensitivity (MIC values in μg/ml) *of Aspergillus fumigatus* isolates to a panel of fungicides belonging to different modes of action.

**Isolate**	**Origin**	**IMA**	**VRC**	**TEB**	**ITC**	**TRB**	**CAR**	**PYR**	**BOS**
BB6-8-B1	D262Y	0.249	0.127	0.572	1.602	0.165	0.843	0.477	-
SS5-2A	D262Y	0.398	0.531	0.318	0.666	0.295	1.619	0.375	0.199
BB1-2.2-B1	Wt	0.398	0.181	0.814	0.301	0.185	1.048	1.110	0.407
PG2-6	D262Y	0.447	0.230	0.198	0.266	0.165	0.843	0.423	-
WT-NL	Wt	0.978	0.342	0.829	1.056	0.338	1.029	2.291	0.321
PG3-3	Wt	1.015	0.531	0.814	0.518	0.332	1.303	1.799	0.517
AF65	Wt	1.141	0.329	0.814	1.413	0.332	1.619	1.413	0.157
PG2-10	Wt	1.283	0.161	2.086	2.241	0.419	1.303	-	-
BB1-3-B9	F46Y/M172V/E427K	1.283	0.857	4.227	10.517	0.263	1.619	1.110	0.321
SS5-7C	F46Y/M172V/E427K	1.620	0.418	1.649	3.401	0.263	1.619	1.413	-
BB6-8-C7	F46Y/M172V/E427K	1.622	0.531	1.466	3.856	0.295	0.609	1.413	-
G1-A9	F46Y/M172V/E427K	1.622	1.088	3.341	17.371	0.332	1.303	1.799	-
UK2-B4	F46Y/M172V/E427K	1.824	0.329	2.970	>22.324	0.471	1.169	0.984	0.407
F5-C6	F46Y/M172V/E427K	2.306	0.531	2.086	8.184	0.419	1.619	1.799	-
AF293	F46Y/M172V/N284T/D255E/E427K	2.915	0.531	1.649	13.516	0.419	1.303	0.538	-
SS10-6A	TR_34_/L98H	2.915	1.381	4.227	>22.324	0.208	1.303	0.231	0.321
SS8-7A	TR_34_/L98H	2.915	2.227	5.348	>22.324	0.208	1.303	2.291	0.407
TR34-NL	TR_34_/L98H	3.156	1.505	6.876	7.219	0.180	1.048	0.872	0.224
08-19-02-10	TR_34_/L98H	3.277	1.754	5.348	>22.324	0.208	1.619	1.110	0.407
F1-C5	TR_34_/L98H	4.142	1.754	2.640	>22.324	0.332	1.619	0.984	0.199
UK5-B5	TR_34_/L98H	4.142	1.754	4.227	>22.324	0.295	>11.464	>20.120	>18.469
RN8-18	TR_34_/L98H	4.142	1.754	4.227	>22.324	0.103	1.048	0.423	0.407
BTCa-1	TR_34_/L98H	5.236	0.857	3.341	4.955	0.595	>11.464	>20.120	-
HPPb-1	TR_34_/L98H	5.236	3.591	5.348	>22.324	0.165	>11.464	>20.120	-
BTTa-1	TR_46_/Y121F/M172I/T289A/G448S	>43.153	>19.120	>17.349	3.000	0.263	>11.464	>20.120	0.199
BKCb-1	TR_46_/Y121F/T289A/S363P/I364V/G448S	>43.153	>19.120	>17.349	1.100	0.116	>11.464	>20.120	5.606
TR46-NL	TR_46_/Y121F/T289A	>43.153	>19.120	>17.349	>22.324	0.286	>11.464	>20.120	0.407
RS3-3	TR_46_/Y121F/T289A	>43.153	>19.120	>17.349	>22.324	0.185	>11.464	>20.120	0.253
WN19-3	TR_46_/Y121F/T289A	>43.153	>19.120	>17.349	>22.324	0.263	>11.464	>20.120	>18.469
WN28-6	TR_46_/Y121F/T289A	>43.153	>19.120	>17.349	>22.324	0.208	>11.464	0.685	0.157

*Isolates ranked according to imazalil sensitivity (low to high MIC values)*.

*IMA (imazalil), VRC (voriconazole), TEB (tebuconazole) and ITC (itraconazole) are azoles, inhibiting 14α-demethylase (sterol biosynthesis); TRB (terbinafine) inhibits squalene-oxidase (sterol biosynthesis); CAR (carbendazim) is a MBC fungicide, inhibiting β-tubulin assembly (cytoskeleton); PYR (pyraclostrobin) is a QoI fungicide, inhibiting respiration (complex III); BOS (boscalid) is a SDHI fungicide, inhibiting respiration (complex II); -, not determined*.

### Resistance to MBC, QoI, and SDHI Fungicides

Insensitivity to carbendazim, pyraclostrobin and boscalid was also measured in a proportion of isolates (Table 4). All six TR_46_ strains (TR46-NL included) were highly resistant to the MBC fungicide carbendazim (MIC > 11.464 μg/ml), with five of them also resistant to the QoI fungicide pyraclostrobin (MIC > 11.464 μg/ml). Two of these strains, BKCb-1 and WN19-3, were also moderately or highly resistant to the SDHI fungicide boscalid with MIC values of 5.606 and >18.469 μg/ml, respectively. Carbendazim, pyraclostrobin and boscalid resistance was also detected in several TR_34_ isolates but not in wild-type and F46Y/M172V/E427K isolates. Cytochrome *b* gene sequence analysis (MW119309 and MW119310) showed the presence of a mutation leading to the replacement of glycine (GGT) by alanine (G*C*T) at codon 143 (G143A) of the protein in five pyraclostrobin resistant strains tested (UK5-B5, HPPb-1, BTTa-1, RS3-3, and WN19-3), all showing MICs >20.120 μg/ml. G143A was not detected in two pyraclostrobin sensitive strains (RN8-18 and 08-19-02-10) with MICs of 0.423 and 1.110 μg/ml, respectively. Six carbendazim resistant isolates tested (HPPb-1, BTTa-1, BKCb-1, RS3-3, WN19-3, and WN28-6), all with MICs >11.464 μg/ml, carried a mutation that resulted in the replacement of phenylalanine (TTC) by tyrosine (T*A*C) at codon 200 (F200Y) of beta-tubulin (MW119311 and MW119312). No target site alterations were found in the sequence of the carbendazim sensitive strain RN8-18 with a MIC of 1.048 μg/ml. Only three out of 20 strains further characterized were insensitive to boscalid. Two strains, UK5-B5 and WN19-3, showed high levels of resistance with MICs >18.469 μg/ml, while strain BKCb-1 with a MIC of 5.606 μg/ml was moderately resistant. Sequencing the SdhB, C and D encoding genes from a boscalid sensitive (RN8-18), moderate resistant (BKCb-1) and highly resistant strain (WN19-3) revealed two different *SdhB* mutations causing alterations at codon 270 of the protein (MW119305, MW119306, and MW119307). SdhB alteration H270Y, histidine (CAC) replaced by tyrosine (*T*AC) was found in WN19-3, while H270R, histidine (CAC) replaced by arginine (C*G*C) was identified in BKCb-1.

### Molecular Characterization of *A. fumigatus* Isolates Using CSP and STR*Af* Typing

CSP typing showed a high level of diversity among the selected isolates (Table 3). Four CSP types, t02^*^, t08, t15, and t19, were detected in F46Y/M172V/E427K strains. In addition to these, we also found additional F46Y/M172V/E427K strains with CSP t07 or t13 as part of this study (data not shown). All these six CSP types are closely related (Figure 4). STR*Af* profiling showed that several markers were conserved (2A, 2C, 4A, and 4B) in all six F46Y/M172V/E427K strains that were further characterized (Table 3). CSP types t02, t04B, and t11 were detected in TR_34_/L98H strains and t01 and t02 in TR_46_/Y121F/T289A strains. The CSP types detected in the TR_34_/L98H and TR_46_/Y121F/T289A strains are different from those detected in F46Y/M172V/E427K strains and cluster together in one or two different groups with t11 separated (Figure 4). Two pairs of strains with identical CYP51A, CSP type, and STR*Af* profile were detected. BB-1-3-B9 and G1-A9 having F46Y/M172V/E427K, CSP t02^*^ and STR*Af* profile [10-14-10-17-13-8-7-5-6] in common, as well as mating type MAT1-1. TR_34_/L98H strains 08-19-02-10 and RN8-18 have STR*Af* profile [25-24-12-84-9-9-8-10-11] and have also CSP t04B and mating type MAT1-2 in common. TR_34_/L98H strains SS10-6 and SSB8-7 carry both CSP t11 and have five out of nine STR*Af* markers identical.

## Discussion

Flower bulb waste, green waste and wood chippings have recently been reported as “hotspots” for azole resistance selection in the Netherlands but more information on other hotspots will be needed to reduce the further selection and spread of fungicide resistant alleles. The aim of this study was to investigate if fungicide applications on cereals can be regarded as a hotspot for azole resistance selection.

None of the 180 strains isolated from soil samples at Broadbalk, taken from either untreated wheat crops (*n* = 90) or from plots sprayed with foliar fungicides (*n* = 90), tested positive for pan-azole resistant TR_34_- or TR_46_-based CYP51A variants. Pan-azole resistant strains were also not detected amongst the 30 strains isolated from Park Grass experiment (permanent grass land). Furthermore, only two out of 418 strains (0.5 %) isolated from soils sampled from commercial wheat fields in France, Germany and the UK were pan-azole resistant. Both strains, one from France (F1-C5) and one from the UK (UK5-B5), carried TR_34_/L98H (Table 3). Straw incorporation did not increase the incidence of pan-azole resistant strains in soil samples taken from winter wheat experiments carried out in Harpenden and Woburn (UK). Three strains [one TR_34_/L98H (RN8-18) and two TR_46_/Y121F/T289A (WN19-3 and WN28-6)] out of 59 isolates (5.1%) were detected in soils from fungicide treated plots without straw incorporation, while only one TR_46_/Y121F/T289A strain (RS3-3) out of 63 isolates (1.6%) was found in soils of fungicide treated plots with straw incorporation. Only two out of 154 strains (1.3%) sampled from grassland, forest and other arable crop soils in different European countries were pan-azole resistant. Both isolates, one from a Dutch corn field (SS10-6A) and one from a sugar beet field in Belgium (SS8-7A), carried TR_34_/L98H. Low frequencies of pan-azole resistant strains between 0 and 4.0% were found in air samples obtained at different urban locations in the Netherlands and the UK. This low background level of pan-azole resistant isolates in air samples is similar to the frequencies of resistant isolates found in soil samples of wheat crops but much lower than the frequencies reported for azole-containing flower bulb waste heaps (6.2–24.5%), where high numbers of spores are present and can be released into the air (Schoustra et al., 2019). We also did not detect an impact of long-term azole-based foliar fungicide applications on the selection of resistant strains in the Broadbalk experiment, comparing treated with untreated plots, and conclude that cereal production is not a hotspot for azole resistance development in *A. fumigatus* as predicted by Gisi (2014).

In addition to wild-type, five different CYP51A variants were found in this study. Regarding azole sensitivity, variant D262Y was equally or less sensitive to azoles in comparison with wild-type isolates. Except itraconazole, for which different resistance mechanisms have been reported, TR_46_/Y121F/T289A isolates showed higher levels or resistance to the azoles voriconazole, imazalil, and tebuconazole than TR_34_/L98H isolates (Table 4). Similar patterns of MIC distributions to itraconazole and voriconazole have also been reported for wildtype, TR_34_/L98H and TR_46_/Y121F/T289A strains using methods described by the European Committee on Antimicrobial Susceptibility Testing (EUCAST) (van Ingen et al., 2015) and the Clinical and Laboratory Standards Institute (CLSI) M38-A2 document (Buil et al., 2018). A TR_46_/Y121F/M172I/T289A/G448S isolate (BTTa-1) and a TR_46_/Y121F/T289A/S363P/I364V/G448S isolate (BKCb-1) were also found in this study. These more complex variants show similarities with the evolution of azole resistance in the plant pathogen *Zymoseptoria tritici*, where a stepwise accumulation of CYP51 mutations, determined by a negative trade-off between enzyme stability through reduced azole binding and enzyme functionality, has been reported to adapt to selection pressure exerted by different azoles entering and dominating the market (Cools and Fraaije, 2013). CYP51A amino acid substitutions Y121F, I364V, and G448S have homologous CYP51 position counterparts in other plant pathogens that have developed azole resistance (Mair et al., 2016). For example, G448S is equivalent to G460S in CYP51B of *Pyrenopeziza brassicae* conferring resistance to different azoles in this fungus (Carter et al., 2014). Mutations equivalent to Y121F and I364V have also evolved in *Z. tritici* (CYP51B Y137F and I381V), conferring resistance to triadimenol and tebuconazole, respectively (Mullins et al., 2011; Cools and Fraaije, 2013). Modeling of the *A. fumigatus* CYP51A protein showed that S363 is one of the key residues anchoring the heme and, therefore, likely to affect azole binding (Fraczek et al., 2011), while other studies showed that I364 forms part of the azole binding pocket (Liu et al., 2016). As CYP51s are less conserved than other fungicide target proteins such as beta-tubulin and cytochrome *b, in vitro* gene modification techniques can be undertaken to establish the precise impact of single and multiple target-site changes on inhibitor binding.

In addition to pan-azole resistant TR_34_ and TR_46_ strains, a proportion of strains isolated from the different soil samples showed high MIC values for itraconazole and, often, also elevated MICs for tebuconazole and, to a lesser extent, imazalil and voriconazole (Table 4). F46Y/M172V/E427K was identified in most of these strains and this variant has been reported for both clinical and environmental strains in Europe and Australia since 2001 (Garcia-Rubio et al., 2018). F46Y/M172V/E427K isolates are generally not considered resistant (Rodriguez-Tudela et al., 2008), but resistant isolates exceeding the EUCAST susceptibility break points for itraconazole (2.0 μg/ml), voriconazole (2.0 μg/ml), and/or posaconazole (0.25 μg/ml) have been reported in other studies (Howard et al., 2009; Garcia-Rubio et al., 2018). One new CSP variant t02^*^ was found in this study. This new type, only found in F46Y/M172V/E427K isolates so far, can be distinguished from t02 by its flanking sequences (Figure 4). Based on the current nomenclature describing 29 different CSP types (Duarte-Escalante et al., 2020), t02 can be renamed as t02A and t02^*^ as t02B. All F46Y/M172V/E427K strains carried closely related CSP types, t02^*^, t08, t13, t15, and t19 (Figure 4), and had identical or similar STR*Af* profiles (Table 3), indicating a separate lineage or cryptic sister species as suggested in other studies using whole genome sequencing (Garcia-Rubio et al., 2018). We recently established that strain IMI 16152 (NRRL 163), isolated in 1911 from chicken lung by C. Thom (Peterson, 1992), is also carrying CYP51A F46Y/M172V/E427K. Because of its presence long before the introduction of azole fungicides in both agricultural and clinical settings in the 1970's and early 1980's (Maertens, 2004; Russell, 2005), respectively, we consider F46Y/M172V/E427K as an example of standing variation rather than acquired resistance.

Acquired resistance against azoles can take place both in patient and in agricultural settings in response to exposure to azole compounds (Hagiwara et al., 2016). The genetic variation measured among TR_34_/L98H and TR_46_/Y121F/T289A isolates has been less in comparison with wild-type isolates indicating single recent origins of the resistant genotypes (Snelders et al., 2008; Chowdhary et al., 2013). Although *A. fumigatus* can undergo asexual, parasexual and sexual stages, some populations, including a lineage harboring TR_34_/L98H isolates, seem to reproduce predominantly asexually (Klaassen et al., 2012). A close association with CSP types and isolates carrying CYP51A TR_34_/L98H (CSP t02, t03, t04A, t04B, and t11) or TR_46_/Y121F/T289A (CSP t01, t02, and t04A) was also observed in other studies (Camps et al., 2012b; Bader et al., 2015). The presence of identical clones in different countries supports clonal expansion of resistant genotypes over long distances by airborne dispersal and/or transport of colonized agricultural produce (Chowdhary et al., 2012; Dunne et al., 2017; Sewell et al., 2019). Two identical clones were found in this study based on STR*Af* profiling (Table 3). One clone represented by isolates BB-1-3-B9 and G1-A9, originating from wheat fields in the UK and Germany, respectively, have also MAT1-1, CSP type t02^*^, and F46Y/M172V/E427K in common, and showed a close resemblance to Danish clinical F46Y/M172V/E427K and TR_120_/F46Y/M172V/E427K strains with only one out of nine STR*Af* markers different (14 instead of 13 at 2B) (Hare et al., 2019). The other clone was formed by isolate RN8-18, isolated from a wheat field in the UK, and reference strain 08-19-02-10, a Dutch environmental isolate from 2008 (Abdolrasouli et al., 2015), with also TR_34_/L98H, CSP t04B, and MAT1-2 in common. TR_34_/L98H strain HPPB-1, isolated from an UK air sample, is very similar to an Australian clinical TR_34_/L98H strain isolated in 2012 (Kidd et al., 2015) with only one STR*Af* marker different (19 for 18 at 2C). A clonal expansion based on an identical STR*Af* profile was also found for TR_46_/Y121F/T289A strain RS3-3, sampled from an UK wheat field, matching with a German clinical TR_46_/Y121F/T289A isolate dating back to 2012 (Steinmann et al., 2015) and several French clinical TR_46_/Y121F/T289A strains in 2013 (Lavergne et al., 2015). With exception of STR*Af* marker 3C (21 instead of 22), one of the two markers for which low levels of instability have recently been reported (De Groot and Meis, 2019), soil isolates from India carrying TR_46_/Y121F/T289A also showed the same profile as RS3-3 (Steinmann et al., 2015).

In addition to azoles, resistance to MBC, QoI, and SDHI fungicides, commonly used in agriculture and, with exception of SDHIs, also for material preservation but not in clinical settings, was also detected in several pan-azole resistant isolates (Table 4). This confirms that *A. fumigatus* as a non-target organism can evolve acquired resistance to agricultural fungicides in the environment. Selection for MBC resistance is currently expected to be minimal for cereal production because the use of this fungicide group as seed treatment or in foliar spray applications in the UK has been very low since 2006 due to resistance development in a range of target pathogens (Hawkins and Fraaije, 2018). All six TR_46_ and three out of nine TR_34_ strains tested (reference strains included) showed high levels of resistance to carbendazim associated with beta-tubulin F200Y, a mutation commonly found in other plant pathogens that have evolved resistance to MBC fungicides after exposure (Hawkins and Fraaije, 2016; Mair et al., 2016). In contrast to beta-tubulin E198A, which is associated with high levels of resistance to carbendazim but sensitivity to the N-phenyl carbamate diethofencarb in several plant pathogens, F200Y confers resistance to both carbendazim and diethofencarb (Koenraadt et al., 1992). While the use of carbendazim as a plant protection product is no longer authorized in the EU, selection for MBC resistance can further occur elsewhere and in areas of crop protection where thiabendazole and thiophanate-methyl, which is degraded to carbendazim, are used. Diethofencarb has been used for treatment of flower bulbs to control *Penicillium* sp. and is still being used in foliar sprays to control diseases like *Botrytis* sp. in fruit and vegetables. Most of MBC-resistant *A. fumigatus* strains were also resistant to pyraclostrobin and this was linked to the presence of cytochrome *b* G143A, a mutation associated with field resistance to QoI fungicides in a range of plant pathogens (Gisi et al., 2002). Only three strains showed insensitivity to boscalid. SdhB H270Y was detected in a strain highly resistant to boscalid (WN19-3; MIC >18.469 μg/ml), whereas SdhB H270Y was detected in a strain with moderate levels of boscalid resistance (BKCb-1; MIC = 5.606 μg/ml). Many different mutations can evolve after exposure to SDHI fungicides in experimental evolution experiments with fungi, including *Z. tritici* (Fraaije et al., 2012), and orthologous mutations to both SdhB H270Y and H270R have been reported in resistant field isolates of several plant pathogens, including *Botrytis* sp. on strawberries and tulips, *Alternaria alternata* on almonds and *Stemphylium botryosum* on asparagus (Sierotzki and Scalliet, 2013; Mair et al., 2016). Broad spectrum azole, MBC, QoI, and SDHI fungicides were introduced into the market in 1973, 1976, 1992, and 2003, respectively (Russell, 2005; Sierotzki and Scalliet, 2013). Isolates with resistance to multiple groups of fungicides have an advantage in habitats where there is abundant growth and sporulation in the presence of different fungicides such as compost and flower bulb waste heaps, and stockpiles of other fungicide-containing plant waste, the so called hotspots (Zhang et al., 2017; Schoustra et al., 2019). Favorable environmental conditions for the sexual stage can occur during composting and new azole resistant genotypes have been detected as well in this environment in the Netherlands. These included TR_46_/Y121F/T289A/I364V, TR_46_/Y121F/M172I/T289A/G448S, a variant reported in the Netherlands in 2010 (Zhang et al., 2017) and recently in Iran (Ahangarkani et al., 2020), and ${\text{TR}}_{46}^{2}$/Y121F/M172I/T289A/G448S and ${\text{TR}}_{46}^{3}$/Y121F/M172I/T289A/T289A/G448S, multiple 46-bp promoter repeat variants (Schoustra et al., 2019). The ${\text{TR}}_{46}^{3}$ variant has also been found in Dutch clinical isolates since 2012 (Zhang et al., 2017). We found low numbers of pan-azole resistant isolates in soils from cereal fields and no new azole-resistant genotypes were identified. However, TR_46_/Y121F/M172I/T289A/G448S and TR_46_/Y121F/T289A/S363P/I364V/G448S, a novel CYP51A variant not reported before, were found in aerosols sampled at two urban locations in the Netherlands, indicating that new pan-azole resistant genotypes are evolving in habitats other than cereals, which can be regarded as a coldspot.

The latest report of a clinical case of infection with an azole resistant *A. fumigatus* strain carrying F46Y/M172V/E427K that acquired a 120-bp tandem repeat during long-term azole treatment, shows that the TR-mediated resistance mechanism is not restricted to environmental isolates only (Hare et al., 2019). In addition, the recent findings of airborne transmission of *A. fumigatus* by patients through coughs and sputum shows that not only spread of resistance mechanisms from environment-to-patient should be considered but also the spread from patient-to-environment and patient-to-patient (Lemaire et al., 2018; Engel et al., 2019). The origin of TR_34_ and TR_46_ based resistance mechanisms might be difficult to determine, however, the evolution of fungicide resistance is a continuous process in both clinical and environment settings where no borders exist for *A. fumigatus*. Monitoring the presence and dynamics of alleles linked with resistance to azole, MBC, QoI, and SDHI in *A. fumigatus* populations will be useful to identify hotspots and coldspots for resistance development in the wider environment. Measures to reduce inoculum build-up, to prevent dispersal and minimize resistance development using tailored fungicide resistance management strategies can then be introduced and validated. A one-health approach, taking into account the relationship between health and disease at the human, animal and environment interfaces, will be needed to tackle the further spread of antifungal resistance and to safeguard the value of azoles for both human health and food security.

## Data Availability Statement

The original contributions presented in the study are included in the article/Supplementary Material, further inquiries can be directed to the corresponding author/s.

## Author Contributions

BF and AM designed the experiments. SA, BF, and SH performed the experiments. BF and SH analyzed the data. BF, AM, and JL participated in writing and/or editing the manuscript. All authors contributed to the article and approved the submitted version.

## Conflict of Interest

The authors declare that the research was conducted in the absence of any commercial or financial relationships that could be construed as a potential conflict of interest.
