# Supplementary material for: The Multi-Fungicide Resistance Status of Aspergillus fumigatus Populations in Arable Soils and the Wider European Environment
Source: Front Microbiol. 2020 Dec 15;11:599233. doi: 10.3389/fmicb.2020.599233 (PMC7770239; doi:10.3389/fmicb.2020.599233)
Supplement: Supplementary file 1 [file Table_1.DOCX]

**Table S1.** Amplification and sequencing of PCR products

| Target | Primers^1^ | Annealing temperature (^o^C) | | Amplicon size (bp) |
| --- | --- | --- | --- | --- |
| Beta tubulin | BTF1: CTCTCCTCCAGCAAATCATCCACA  BTR1: GCCGAGAGTTAGTACACGTACTCT  *BTFS: GACAACTTCGTCTTCGGCCAGT*  *BTRS: CTCCTCACGGATCTTGGAGATC* | | 60 | 1971 |
| CSP | CSPF1: TTGGGTGGCATTGTGCCAA  CSPR1: GGAGGAACAGTGCTGTTGGTGA | | 62 | 567-711^2^ |
| CYP51A | 51AF1: GGAGAAGGAAAGAAGCACTCT  51AR1: CTGTCTCACTTGGATGTG  *51AFS: CCTCTATCATGACCTGGACA*  *51ARS: ATGAGCAGCATCTCGCTTCT* | | 53 | 2040^3^ |
| CYP51B | 51BF2: CCTCTAGGCGAGACACTCAGCCTA  51BR2: GCGAACTGCCAACAAGACGGCAGA  *51BFS: CCACATTCGCAGAGTTGTAC*  *51BRS: GCCCAGGGTAACATGAAGTT* | | 65 | 1919 |
| Cytochrome *b* | CBF1: CAGGTGTTACATTAGCTATGCACTATACAC  CBR1: CGGAACAATAGCAGGTGGAG | | 62 | 680 |
| Mating type | AFM1: CCTTGACGCGATGGGGTGG  AFM2: CGCTCCTCATCAGAACAACTCG  AFM3: CGGAAATCTGATGTCGCCACG | | 65 | 438 & 834^4^ |
| SdhB | SBF1: ATGGCCGCTCTTCGT  SBR1: CTAATGGGTAGCAAGCATCTTC | | 58 | 1016 |
| SdhC | SCF1: ATGATCTCTCAGAAGGTTGCTCA  SCR1: TCACAGCAAAGCAAGAGTGAG | | 60 | 804 |
| SdhD | SDF1: ATGGCTTCGATTGCG  SDR1: CTATGCCTTCCACACCC | | 58 | 924 |

^1^ PCR primers as described by Hurst *et al*., 2009 (CSP), Diaz-Guerra *et al*., 2003 and Spiess *et al*., 2012 (CYP51A) and Paoletti *et al*., 2005 (mating type MAT1-1 and MAT1-2), additional sequencing primers shown in italics; ^2^ CSP amplicon size depends on tandem repeat numbers, between 4 and 16 according Duarte-Escalante *et al*., 2020; ^3^ CYP51A amplicon size without TR promoter inserts; ^4^ 834 bp for MAT1-1 and 438 bp for MAT1-2

**REFERENCES**

Diaz-Guerra, T. M. E., Mellado, E., Cuenca-Estrella, M., and Rodriguez-Tudela, J. L. (2003). A point mutation in the 14-alpha sterol demethylase gene cyp51A contributes to itraconazole resistance in Aspergillus fumigatus. *Antimicrob.* *Agents Chemother*. 47, 1120–1124. doi: 10.1128/AAC.47.3.1120-1124.2003

Hurst, S. F., Kidd, S. E., Morrissey, C. O., Snelders, E., Melchers, W. J., Castelli, M. V., et al. (2009). Interlaboratory reproducibility of a single-locus sequence- based method for strain typing of *Aspergillus fumigatus*. *J. Clin. Microbiol*. 47, 1562–1564. doi: 10.1128/JCM.00124-09

Paoletti, M., Rydholm, C., Schwier, E. U., Anderson, M. J., Szakacs, G., Lutzoni, F., et al. (2005). Evidence for sexuality in the opportunistic fungal pathogen *Aspergillus fumigatus. Curr. Biol*. 15, 1242–1248. doi: 10.1016/j.cub.2005.05.045

Spiess, B., Seifarth, W., Merker, N., Howard, S. J., Reinwald, M., Dietz, A., et al. (2012). Development of novel PCR assays to detect azole resistance-mediating mutations of the Aspergillus fumigatus cyp51A gene in primary clinical samples from neutropenic patients*. Antimicrob. Agents Chemother*. 56, 3905–3910. doi: 10.1128/AAC.05902-11
